# A Bioinformatic Algorithm based on Pulmonary Endoarterial Biopsy for Targeted Pulmonary Arterial Hypertension Therapy

**DOI:** 10.2174/18743064-v17-230927-2023-9

**Published:** 2023-08-17

**Authors:** Abraham Rothman, David Mann, Jose A. Nunez, Reinhardt Tarmidi, Humberto Restrepo, Valeri Sarukhanov, Roy Williams, William N. Evans

**Affiliations:** 1 Children’s Heart Center Nevada, 3131 La Canada, Suite 230, Las Vegas, NV 89169, USA; 2 Department of Pediatrics, Division of Pediatric Cardiology, Kirk Kerkorian School of Medicine at UNLV, 2040 W. Charleston Blvd Ste. 402, Las Vegas, NV 89109, USA; 3 Vascular Biosciences, 72 Santa Felicia Drive, Goleta, CA, 93117, USA; 4 College of Engineering, University of California, Santa Barbara, Lagoon Rd, Santa Barbara, CA 93106, USA; 5 Institute of Genomic Medicine, University of California, San Diego, 9500 Gilman Drive #0761, La Jolla, CA 92093, USA

**Keywords:** Animal research, Bioinformatic algorithm, Endoarterial biopsy, Pharmacogenomics, Pharmacological therapy, Pulmonary hypertension

## Abstract

**Background:**

Optimal pharmacological therapy for pulmonary arterial hypertension (PAH) remains unclear, as pathophysiological heterogeneity may affect therapeutic outcomes. A ranking methodology based on pulmonary vascular genetic expression analysis could assist in medication selection and potentially lead to improved prognosis.

**Objective:**

To describe a bioinformatics approach for ranking currently approved pulmonary arterial antihypertensive agents based on gene expression data derived from percutaneous endoarterial biopsies in an animal model of pulmonary hypertension.

**Methods:**

We created a chronic PAH model in Micro Yucatan female swine by surgical anastomosis of the left pulmonary artery to the descending aorta. A baseline catheterization, angiography and pulmonary endoarterial biopsy were performed. We obtained pulmonary vascular biopsy samples by passing a biopsy catheter through a long 8 French sheath, introduced *via* the carotid artery, into 2- to 3-mm peripheral pulmonary arteries. Serial procedures were performed on days 7, 21, 60, and 180 after surgical anastomosis. RNA microarray studies were performed on the biopsy samples.

**Results:**

Utilizing the medical literature, we developed a list of PAH therapeutic agents, along with a tabulation of genes affected by these agents. The effect on gene expression from pharmacogenomic interactions was used to rank PAH medications at each time point. The ranking process allowed the identification of a theoretical optimum three-medication regimen.

**Conclusion:**

We describe a new potential paradigm in the therapy for PAH, which would include endoarterial biopsy, molecular analysis and tailored pharmacological therapy for patients with PAH.

## 1 INTRODUCTION

Pulmonary arterial hypertension (PAH) is accompanied by significant morbidity and mortality [1, 2]. Currently approved PAH therapeutic agents mainly target three molecular pathways [3]; nevertheless, evidence-based methodologies for individualizing therapy are lacking. A method for guiding therapy could be based on the identification of specific pulmonary vascular genetic dysregulation in patients with PAH [4], coupled with pharmacogenomic interaction data between pulmonary arterial antihypertensive agents and pulmonary vascular dysregulated genes. We describe a bioinformatics approach for ranking currently approved pulmonary arterial antihypertensive agents based on gene expression data derived from percutaneous endoarterial biopsies in an animal model of pulmonary hypertension.

## 2 METHODS

Chronic PAH was created in 4 Micro Yucatan female swine by surgical anastomosis of the left pulmonary artery (LPA) to the descending aorta [5]. The mean body weight was 22.4 ± 5.3 kg, and the mean age at surgery was 7.3 ± 2.7 months. An institutional animal research committee at the University of Nevada Las Vegas approved the protocol. Anesthesia was induced and maintained with inhaled isoflurane (Baxter Healthcare Co. Deer Field, IL, USA) as described previously [5]. A baseline right-sided cardiac catheterization with pulmonary angiography was performed through a sheath in the right internal jugular vein. The biopsy procedure was performed as described previously [6, 7]. To obtain biopsies, an 8F long sheath was wedged in 2- to 3-mm peripheral pulmonary arteries. At each procedure, two samples were obtained for RNA analysis. Catheterization with aortic and pulmonary artery pressure measurement, angiography, and endoarterial biopsies was performed through an 8F sheath in the carotid artery at days 7, 21, 60, and 180 after surgery. Briefly, the biopsy catheter was advanced through the delivery sheath into the distal vessel. The biopsy catheter was changed from closed to open configuration, exposing a window in the distal steel end of the internal tube. The vacuum was turned on. The trigger in the biopsy catheter was activated, resulting in the outer tube of the biopsy catheter advancing over the inner tube, cutting the tissue sample. The catheter was removed from the sheath and the biopsy piece was retrieved. Angiograms in distal pulmonary artery branches were performed before and after biopsy. To create the shunt model, a left thoracotomy was performed in the fourth intercostal space. The LPA was ligated at its origin from the pulmonary trunk. The descending thoracic aorta was clamped, and a window was created in its medial aspect with a 4.5 mm punch. An end-to-side anastomosis was created. The chest was closed. No chest tubes were placed. Postoperative care was as described previously [5]. Pulmonary arterial pressures were normal in the early biopsy time points and at near systemic level on day 180 [5]. Pulmonary arterial mural changes included progressive neo-intimal formation, endothelial abnormalities and medial thickening as the PAH advanced; findings consistent with significant PAH and described in one of our previous publications [5]. Animals were anesthetized and intubated for each procedure, allowed to recover, and re-instrumented for repeat procedures until day 180, when they were euthanized and subjected to pathologic analysis [5]. For RNA microarray analysis, biopsy samples were placed in RNAlater™ and analyzed by GeneChip^®^ Porcine Genome Array, Thermo Fisher Scientific, which provides comprehensive coverage of the Sus scrofa transcriptome, containing 23,937 probe sets for 20,201 genes. The sequence information was selected from UniGene Build 28, GenBank^®^ mRNAs, and GenBank^®^ porcine mitochondrial and rRNA sequences. Specimens were homogenized using QIAshredder columns in a FastPrep FP120 Homogenizer. RNA was isolated using RNeasy Mini columns and quantified initially by UV spectrophotometry and more definitively by capillary electrophoresis on an Agilent 2100 Bioanalyzer.

A list of drugs approved for the treatment of PAH were compiled. A literature review of the genes affected by treatment with each individual PAH drug was conducted, and a set of PAH drug target genes was created. This set of genes associated with specific PAH drugs was cross-referenced with data generated from the GeneChip^®^ Porcine Genome Array. From these data sets, a new set of genes was generated: Genes affected by PAH drugs for which quantitative information from the GeneChip^®^ Porcine Genome Array was available. A literature review of the PAH drug-associated genes was conducted to identify which genes were clinically relevant in the pathology of PAH. A final list of genes was generated. The specific drugs chosen for the ranking method were included based on available gene data from the GeneChip^®^ Porcine Genome Array.

For each affected gene, the fold change in expression from baseline was calculated on days 7, 21, 60, and 180 post-surgery, based on data derived from the GeneChip^®^ Porcine Genome Array. To determine drug scores, the fold change of each affected gene was then multiplied by an “alignment factor” of -1 if the drug-gene interaction followed gene expression associated with pathology or if the drug-gene interaction reduced expression of a gene known to be upregulated as a compensatory mechanism to alleviate pathology (such as upregulation of prostaglandin synthase), or +1 if the drug-gene interaction counteracted gene expression associated with pathology or upregulated compensatory genes. The final score for a drug at a given time point was calculated by averaging the modified fold changes for all affected genes known to interact with the drug of interest. The calculated scores then determined the final rankings of the drugs, with higher scores indicating larger magnitudes of drug-gene interactions that counteracted gene expression associated with pathology. The equation used for calculating scores, a modified mean, is shown below:


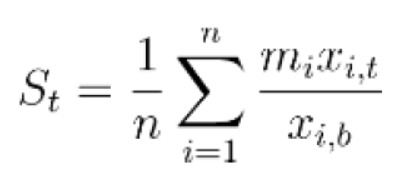


where *St* is the drug’s score on day *t*, *n* is the total number of genes affected by the drug for which data were available (derived from Table **2**), *mi* is the alignment factor of -1 or 1 associated with the drug’s effect on the expression of gene *i*, *xi,t* is the raw gene expression of gene *i* on day *t*, and *xi,b* is the baseline gene expression of gene *i*.

## 3 RESULTS

We examined the Food and Drug Administration (FDA) approved PAH therapeutic agents; however, we limited the analysis to 6 medications that had sufficient associated genetic expression data: ambrisentan, bosentan, iloprost, macitentan, sildenafil and treprostinil. Table **1** lists genes identified from the literature and the porcine genome array, relevant to PAH pathology and affected by the therapeutic agents. For each of the therapeutic agents, the gene-expression fold change at each of the four data acquisition periods (not shown) was utilized to rank the therapeutic agent at the specific time points. Table **2** reveals the calculated rank scores for all six PAH medications, derived from GeneChip^®^ Porcine Genome Array gene expression data. As an example from Table **2**, based on the ranking of ambrisentan, along with the other two endothelin receptor antagonists (bosentan and macitentan), macitentan would be the preferred choice on day 21; whereas ambrisentan would be the preferred choice at day 180.

The ranked calculated scores, for all agents from all experimental time periods, are shown in Table **2** and Fig. (**1**). The prostacyclins, treprostinil and iloprost had similar rankings on days 7 and 60; however, treprostinil had higher rankings on days 21 and 180. Sildenafil was the only phosphodiesterase 5 inhibitor analyzed, and it ranked high on day 180. Table **3** demonstrates the highest-scoring triple therapy at each time point, based on the scores from Table **2**.

## 4 DISCUSSION

Despite advancements in PAH pharmacological therapy, long-term outcomes remain suboptimal [8]. FDA approved medications primarily target three molecular pathways [3]. Additional agents are under investigation, with some designed to affect alternative molecular pathways [3, 4]. Further, multiple genes are dysregulated in PAH [3, 4], coupled with the complexity of temporally related specific gene dysregulations over the course of PAH [9]. Currently, pharmacological therapy comprises a regimen of a combination of agents. often lacking specific evidence-based guidance. A method to assess pathological gene dysregulations over time along with associated pharmacogenomic interactions of selected medications might allow for tailored therapy.

In this manuscript, our aim was to describe a new paradigm in the therapy of PAH, which consists of three parts. The first part is to obtain a biopsy of pulmonary arterial wall. The second part entails performing molecular analysis on the biopsy piece. We chose to use a porcine gene chip microarray for our study because it matched out model; however, other techniques, such as RNA-Seq could have been used and would be more desirable in human studies. The third part of our method consists of ranking medications (in this case, ones approved for the treatment of PAH) to identify individual drugs or combinations of drugs that could be used to target molecular dysregulation.

Past studies have investigated findings of gene dysregulation in PAH patients through analysis of fresh peripheral blood mononuclear cells or whole blood samples [10]. Nevertheless, an analysis based on pulmonary vascular tissue would be preferable. Although not yet clinically available, we previously demonstrated that pulmonary endoarterial tissue can be obtained successfully and safely in experimental animal models [5-7, 9]. Currently, guided therapy *via* ranking gene dysregulation has a role in the selection of therapeutic agents for neoplasms [11, 12].

The use of combination therapy is now considered the standard of care in the treatment of PAH. Maximal therapy consists of an agent each affecting the nitric oxide, endothelin and prostacyclin pathways. In our study, the analysis of optimal triple therapy revealed differences in the choice of drug, which depended on the time point in the progression of PAH that was tested. This optimal therapy remains theoretical because we did not test it in an animal model. In addition, knowledge about whether drugs in the same class cause identical, similar or different effects on gene expression remains incomplete. Nonetheless, we speculate that patients will also have different molecular pathways expressed at different “stages” of their disease, requiring different drug combinations. Logically, as more molecular pathways associated with PAH are discovered, the analysis would add drugs that are known to affect those pathways. This could include drugs that are currently approved or utilized in other diseases. Therefore, analysis of genetic changes on the pulmonary arterial biopsy samples has the potential of optimizing individualized PAH combination therapy. We intend to extend this proof of concept to guide medical management of PAH patients using this biopsy-based bioinformatics drug ranking method.

This study has several limitations. We used only female Micro Yucatan pigs for ease of animal care and their genetic dysregulation in the arterial wall may be different from males or from other species. Our main aim in this study was more to describe a methodology, rather than the preferred way to target the molecular abnormalities in PAH. The endoarterial biopsy technique obtained samples from 2-3 mm distal pulmonary arteries; however, it is unclear that gene dysregulation findings in the biopsied distal pulmonary arteries are similar throughout the pulmonary vasculature. Further, the principal target of prostacyclins, prostacyclin receptors (PTGIR/PGI2/IP), were not included in the analysis due to a lack of prostacyclin-related gene data in the GeneChip^®^ Porcine Genome Array. The resulting lack of data likely had a significant effect on the rankings of the prostacyclin family of drugs. Moreover, the ranking ethodology relied on reports of dysregulated genes from animal or *in vitro* PAH models that analyzed pulmonary tissue or cultured pulmonary cells, rather than endoarterial biopsy tissue. Additionally, we did not utilize data from human pharmacogenomic studies that included dysregulated genes that were not part of our analysis. To account for potential biases in favor of agents more extensively studied than others, we used a mean score for all genes associated with a particular medication in the ranking methodology. Finally, our analysis was limited to 6 drugs. Other FDA-approved PAH medications were not included due to a lack of target gene data from the GeneChip^®^ Porcine Genome Array.

## CONCLUSION

In conclusion, this study demonstrates a new methodology for PAH therapy. It combines a pulmonary endoarterial biopsy technique with pulmonary vascular genetic expression studies to generate a therapeutic agent ranking system upon which tailored PAH therapy might be based. The optimal molecular testing technique remains to be determined. Human studies will be necessary to demonstrate the utility of this approach.

## AUTHORS’ CONTRIBUTIONS

AR and DM: Concept, design, acquisition, and analysis of data, draft the article, critically revise, and approve the final version. Both contributed equally to this manuscript. HR: Acquisition and analysis of data, draft the article, critically revise it, and approve the final version. JAN and RT: Acquisition of data from the literature, data analysis, critically revise it, and approve final version. RW: Analysis of genetic data from the biopsy samples, critically revise and approve final version. WNE and VS: Acquisition of data, critically revise article and approve final version.

## Figures and Tables

**Fig. (1):**
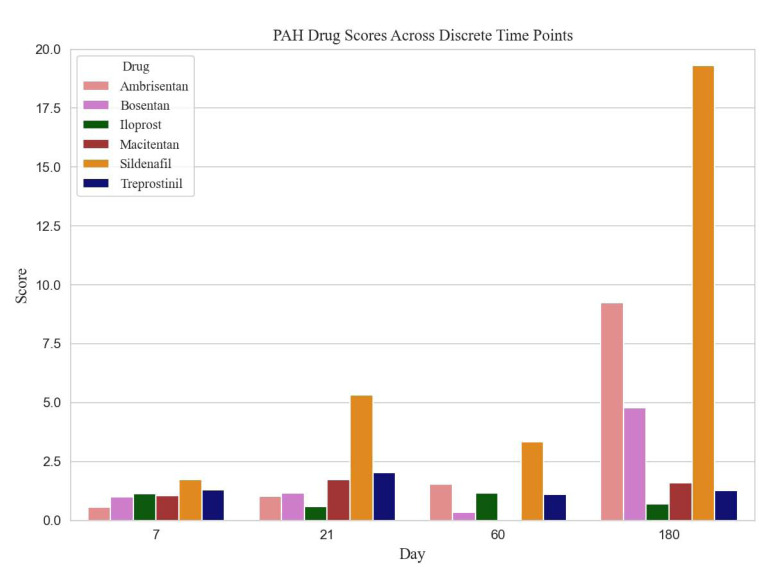
Scores of the six drugs tested in the four time-points.

**Table 1:** Genes clinically relevant to PAH affected by a specific PAH drug and the effect of the drug on the gene.

**Drug**	**Gene Name/Ref**	**Gene ID**	**Mechanism of Action/Ref**
**Ambrisentan**	C-C Motif Chemokine Ligand 19 [13]	CCL19	Inhibitor [14]
	C-C Motif Chemokine Ligand 2 [14-17]	CCL2	Inhibitor [14]
	C-C Motif Chemokine Ligand 5 [18, 19]	CCL5	Inhibitor [14]
	Endothelin 1 [20-22]	EDN1	Inhibitor [20]
	Endothelin Receptor Type A [20, 21, 23]	EDNRA	Inhibitor [20, 24]
	Interleukin 6 [14, 24, 25]	IL6	Inhibitor [14]
	Nitric Oxide Synthase 3 [20]	NOS3	Inducer [20]
**Bosentan**	C-C Motif Chemokine Ligand 19 [13]	CCL19	Inhibitor [14]
	C-C Motif Chemokine Ligand 2 [15-17]	CCL2	Inhibitor [14]
	C-C Motif Chemokine Ligand 5 [18, 19]	CCL5	Inhibitor [14]
	Collagen Type I Alpha 1 Chain [20-28]	COL1A1	Inhibitor [26]
	Endothelin 1 [20-22]	EDN1	Inhibitor [21]
	Endothelin Receptor Type A [20, 21, 23]	EDNRA	Inhibitor [21]
	Endothelin Receptor Type B [13, 29]	EDNRB	Inhibitor [30]
	Fibronectin 1 [26]	FN1	Inhibitor [26, 31]
	Follistatin [26]	FST	Inhibitor [26]
	Interleukin 6 [25]	IL6	Inhibitor [14, 32]
	Galectin 3 [26]	LGALS3	Inhibitor [26]
	Inhibin Subunit Beta A [26]	INHBA	Inhibitor [26]
	Matrix Metallopeptidase 2 [33, 34]	MMP2	Inhibitor [34]
	Natriuretic Peptide A [26]	NPPA	Inhibitor [26]
	Natriuretic Peptide B [26]	NPPB	Inhibitor [26]
	Prostaglandin I2 Synthase [35]	PGIS	Inhibitor [35]
	TIMP Metallopeptidase Inhibitor 1 [26, 34]	TIMP1	Inhibitor [26, 34]
**Macitentan**	Collagen Type I Alpha 1 Chain [26-28]	COL1A1	Inhibitor [26]
	Endothelin Receptor Type A [20, 21, 23]	EDNRA	Inhibitor [36, 37]
	Endothelin Receptor Type B [23, 29]	EDNRB	Inhibitor [36, 37]
	Fibronectin 1 [26]	FN1	Inhibitor [26]
	Follistatin [26]	FST	Inhibitor [26]
	Galectin 3 [26]	LGALS3	Inhibitor [26]
	Inhibin Subunit Beta A [26]	INHBA	Inhibitor [26]
	Matrix Metallopeptidase 2 [33, 34]	MMP2	Inhibitor [33]
	Matrix Metallopeptidase 9 [33]	MMP9	Inhibitor [33]
	Natriuretic Peptide A [26]	NPPA	Inhibitor [26]
	Natriuretic Peptide B [26]	NPPB	Inhibitor [26]
	TIMP Metallopeptidase Inhibitor 1 [26, 34]	TIMP1	Inhibitor [26]
**Sildenafil**	Angiopoietin 1 [38]	ANGPT1	Inhibitor [38]
	Angiotensin II Receptor Type 1 [38]	AT-1	Inhibitor [38]
	Angiotensin II Receptor Type 2 [38]	AT-2	Inhibitor [38]
	Endothelin 1 [20-22]	EDN1	Inhibitor [38]
	Endothelin Converting Enzyme 1 [25]	ECE1	Inhibitor [38]
	Endothelin Receptor Type B [38]	EDNRB	Inhibitor [38]
	Nitric Oxide Synthase 2 [38]	NOS2	Inhibitor [38]
	Nitric Oxide Synthase 3 [38]	NOS3	Inhibitor [38]
	Phosphodiesterase 5A [38]	PDE5A	Inhibitor [38]
	Vascular Endothelial Growth Factor A [32, 38]	VEGFA	Inhibitor [38]
**Iloprost**	Collagen Type I Alpha 1 Chain [26-28]	COL1A1	Inhibitor [28]
	Collagen Type 3 Alpha 1 Chain [27, 28]	COL3A1	Inhibitor [28]
	Cellular Commun. Network Factor 2 [28]	CCN2/CTGF	Inhibitor [28]
	Inhibitor of DNA Binding 1 [39]	ID1	Inducer [39]
	Interleukin 6 [32]	IL6	Inhibitor [40]
	Matrix Metallopeptidase 9 [33]	MMP9	Inducer [28]
	Transforming Growth Factor Beta 1 [36, 41, 42]	TGFB1	Inhibitor [42]
	Tumor Necrosis Factor [32]	TNF	Inhibitor [40]
**Treprostinil**	Collagen Type I Alpha 1 Chain [26-28]	COL1A1	Inhibitor [27, 41]
	Collagen Type III Alpha 1 Chain [27, 28]	COL3A1	Inhibitor [27]
	Fibronectin 1 [26]	FN1	Inhibitor [41]
	Inhibitor of DNA Binding 1 [39]	ID1	Inducer [39]
	Interleukin 6 [32]	IL6	Inhibitor [40]
	Peroxisome Prolif. Activated Receptor Alpha [43]	PPARA	Inducer [43]
	Peroxisome Prolif. Activated Receptor Delta [44]	PPARD	Inducer [44]
	Peroxisome Prolif. Activ. Receptor Gamma [45]	PPARG	Inducer [46]
	TIMP Metallopeptidase Inhibitor 1 [28, 47]	TIMP1	Inhibitor [47]
	Transforming Growth Factor Beta 1 [41, 42, 47]	TGFB1	Inhibitor [41, 47]
	Tumor Necrosis Factor [32]	TNF	Inhibitor [40]

**Table 2:** Calculated rank scores for all six PAH medications, derived from GeneChip^®^ porcine genome array gene expression data.

**Drug**	**Category**	**Day 7 Score**	**Day 21 Score**	**Day 60 Score**	**Day 180 Score**
**Ambrisentan**	Endothelin Receptor Antagonist	0.55	1.03	1.55	9.25
**Bosentan**	Endothelin Receptor Antagonist	1.00	1.16	0.34	4.78
**Macitentan**	Endothelin Receptor Antagonist	1.04	1.73	-0.02	1.58
**Sildenafil**	Phosphodiesterase 5 Inhibitor	1.72	5.33	3.33	19.30
**Iloprost**	Prostacyclin	1.13	0.59	1.17	0.68
**Treprostinil**	Prostacyclin	1.28	2.02	1.10	1.27

**Table 3:** Optimal triple therapy regimen, based on the highest score for each time point.

**Day**	**Prostacyclin**	**PDE5 Inhibitor**	**ERA**
**7**	Treprostinil	Sildenafil	Macitentan
**21**	Treprostinil	Sildenafil	Macitentan
**60**	Iloprost	Sildenafil	Ambrisentan
**180**	Treprostinil	Sildenafil	Ambrisentan

## Data Availability

The datasets used and/or analyzed during the current study are available from the corresponding author [A.R] upon reasonable request.

## LIST OF ABBREVIATIONS

PAH: Pulmonary Arterial Hypertension
FDA: Food and Drug Administration
LPA: Left Pulmonary Artery
